# Adenovirus Vectors Target Several Cell Subtypes of Mammalian Inner Ear* In Vivo*

**DOI:** 10.1155/2016/9409846

**Published:** 2016-12-28

**Authors:** Yilai Shu, Yong Tao, Wenyan Li, Jun Shen, Zhengmin Wang, Zheng-Yi Chen

**Affiliations:** ^1^Department of Otolaryngology, Harvard Medical School and Eaton-Peabody Laboratories, Massachusetts Eye & Ear Infirmary, Boston, MA 02114, USA; ^2^Department of Otolaryngology-Head and Neck Surgery, Eye and ENT Hospital, Shanghai Medical College, Fudan University, Shanghai, China; ^3^Key Laboratory of Hearing Medicine, National Health and Family Planning Commission, Shanghai, China; ^4^Department of Pathology, Brigham and Women's Hospital, Harvard Medical School, Boston, MA 02115, USA; ^5^Laboratory for Molecular Medicine, Partners Personalized Medicine, Cambridge, MA 02139, USA

## Abstract

Mammalian inner ear harbors diverse cell types that are essential for hearing and balance. Adenovirus is one of the major vectors to deliver genes into the inner ear for functional studies and hair cell regeneration. To identify adenovirus vectors that target specific cell subtypes in the inner ear, we studied three adenovirus vectors, carrying a reporter gene encoding green fluorescent protein* (GFP)* from two vendors or with a genome editing gene Cre recombinase* (Cre)*, by injection into postnatal days 0 (P0) and 4 (P4) mouse cochlea through scala media by cochleostomy* in vivo*. We found three adenovirus vectors transduced mouse inner ear cells with different specificities and expression levels, depending on the type of adenoviral vectors and the age of mice. The most frequently targeted region was the cochlear sensory epithelium, including auditory hair cells and supporting cells. Adenovirus with* GFP* transduced utricular supporting cells as well. This study shows that adenovirus vectors are capable of efficiently and specifically transducing different cell types in the mammalian inner ear and provides useful tools to study inner ear gene function and to evaluate gene therapy to treat hearing loss and vestibular dysfunction.

## 1. Introduction

Irreversible hair cell loss is a major cause of permanent sensorineural hearing loss with no effective treatment. Pathogenic variants in hundreds of genes are responsible for many forms of hereditary hearing loss. The development of strategies for hair cell regeneration and for gene delivery has become a major focus in the search for potential therapeutic approaches to restoring hearing [1–3].

Lower vertebrates including birds and fish can regenerate hair cells throughout life after hair cell loss by two mechanisms. First, inner ear supporting cells and remaining hair cells may reenter the cell cycle and differentiate into new hair cells. Second, surrounding cells located under the lost hair cells may also directly transdifferentiate into new hair cells [4–6]. However, the mammalian inner ear has lost the capacity to regenerate hair cells spontaneously. One strategy to regenerate hair cells in mammals to restore hearing is to induce surrounding cells especially supporting cells to transdifferentiate into hair cells directly. Another approach is to induce remaining hair cells or supporting cells to reenter the cell cycle and for supporting cells to further differentiate to hair cells [1, 7]. Either approach requires efficient delivery of genes necessary for the induction of these processes into mammalian inner ear cells.

The inner ear is a particularly attractive organ for targeted gene therapy, because vectors can be locally delivered to the enclosed structure, which significantly reduces systemic side effects. One of the major hurdles to achieve hair cell regeneration or gene correction by gene therapy is the lack of efficient and specific vehicle to deliver genes into mammalian inner ear cells. Adenovirus (Ad) and Adeno-associated virus (AAV) are the most common vectors used for inner ear gene delivery. Both have been used to successfully transfer functional genes into the mammalian inner ear for gene therapy [8–23]. Ad vector is a good choice due to its high transfection efficiency in diverse tissues and cell types, with high level of expression soon after infection. Furthermore, Ad vector has low immunogenicity and toxicity [15–23]. Comparing to AAV vectors, Ad vectors have the capacity to accommodate larger inserts. For example, the most commonly used adenovirus vectors, which are E1/E3 deletion mutants, allow the insertion of up to 10 kb of foreign DNA into the viral vector genome, while an AAV can only carry up to 4.7 kb of foreign DNA [13, 24].

Previous studies have demonstrated the ability of Ad vectors to transduce cochlear hair cells and supporting cells [5–13]. In mammalian inner ear, transduction by Ad vectors is organ (cochlea versus vestibule), cell type (inner hair cells (IHCs), outer hair cells (OHCs), and supporting cells (SCs)), region (base, middle, and apical turns), and age dependence. The transduction patterns of Ad vectors in the inner ear vary. In order to use Ad vectors to effectively deliver genes into specific inner ear cell subtypes, it is important to characterize the transduction patterns of viral vector subtypes under various experimental conditions, including animal age, route of inoculation, viral preparations, volume, and number of viral particles.

To identify commercially available Ad vectors for their inner ear delivery patterns, we analyzed Ad vectors carrying a GFP from Baylor College of Medicine (Ad-GFP-Baylor) and from Vector Biolabs (Ad-GFP-VB) and an Ad vector carrying GFP linked with a genome editing gene Cre recombinase (Cre) from Baylor College of Medicine (Ad-Cre-GFP-Baylor)* in vivo* for their potential for inner ear gene delivery in P0 and P4 mouse cochleae.

## 2. Material and Methods

### 2.1. Ad Vectors

We obtained three commercially available Ad vectors: Ad-GFP-Baylor (Baylor College of Medicine, Houston, TX, USA), Ad-GFP-VB (Vector Biolabs, Malvern, PA, USA), and Ad-Cre-GFP-Baylor (Baylor College of Medicine, Houston, TX, USA). The titers of Ad-GFP-Baylor, Ad-GFP-VB, and Ad-Cre-GFP-Baylor were 2.5 × 10^10^–5 × 10^11^ plaque-forming unit (pfu)/ml, 1 × 10^10^ pfu/ml, and 1.7 × 10^11^ pfu/ml, respectively. We consider the titer of Ad-GFP-Baylor as 10 × 10^10^ pfu/ml for dilution. We diluted all Ads to 1 × 10^10^ pfu/ml with storage buffer according to the vectors instructions from two vendors for microinjection.

### 2.2. Microinjection of Ad Vectors into Mouse Inner Ear

P0 and P4 CD1 mice (Charles River Laboratory, Wilmington, MA, USA) were used for Ad-Cre-GFP-Baylor, Ad-GFP-Baylor, and Ad-GFP-VB injection, according to protocols approved by the Massachusetts Eye & Ear Infirmary IACUC committee. Mice were anesthetized by lowering their temperature on ice. Cochleostomies were performed by making an incision behind the right ear to expose the cochlea. Glass micropipettes (WPI, Sarasota, FL, USA) held by a Nanoliter Microinjection System (WPI, Sarasota, FL, USA) were used to deliver the Ad into the scala media, which allows access to inner ear cells. A total volume of ~0.2 *µ*L was injected per cochlea on the right side and the release was controlled by a micromanipulator at the speed of 3 nL/sec. The left cochlea was left intact as an internal control.

### 2.3. Immunofluorescence and Quantification

Four days after injection, mice were sacrificed and cochleae were harvested by standard protocols [1, 17]. For whole-mount immunofluorescence, primary antibodies against HC (MYO7A, #25-6790, Proteus Biosciences) and SC (SOX2, #sc-17320, Santa Cruz Biotech) markers and fluorescent-labeled secondary antibodies (Invitrogen) were used following a previously described protocol [1]. To quantify the proportion of GFP positive cells after Ad injection, we counted the number of GFP positive IHCs, OHCs, and SCs, which were then divided by the total number of IHCs, OHCs, and SCs, respectively, in a region spanning 200 *µ*m in the apical, middle, or basal turn of the cochlea.

## 3. Results

**Figure 1:**
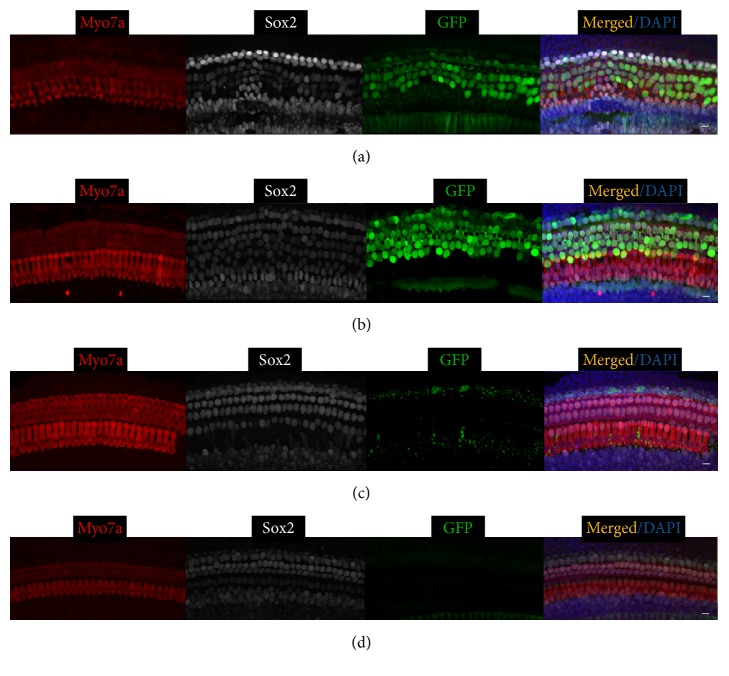
Ad-Cre-GFP-Baylor transduces supporting cells when injected into mouse cochlear at P0. Representative confocal images of whole-mount fluorescent immunolabeling of cochlea injected at P0 to illustrate the basal (a), middle (b), and apical turns (c), as compared to the contralateral uninjected middle turn of cochlea (d). Myo7a labels hair cells, and Sox2 labels supporting cells. Ad-Cre-GFP-Baylor mainly transduces supporting cells in basal and middle turns. Scale bars: 10 *μ*m.

**Figure 2 fig2:**
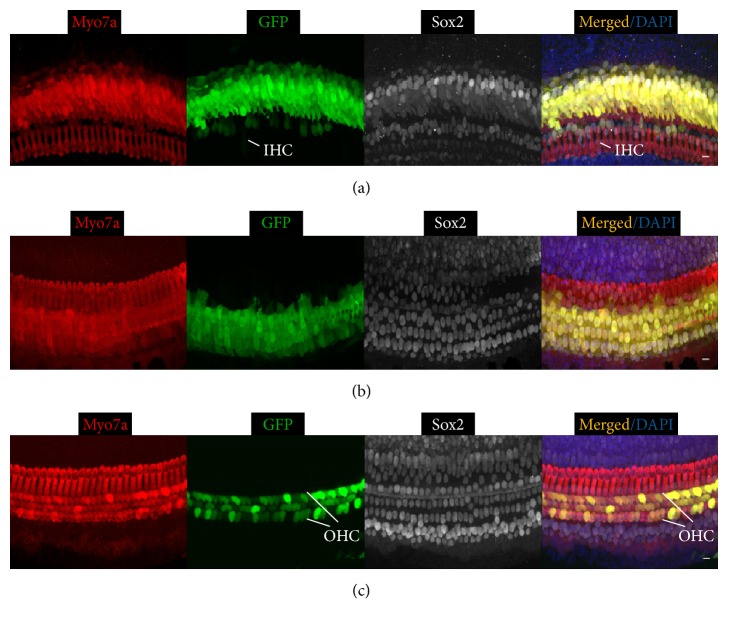
Ad-GFP-Baylor transduces diverse cochlear cell types when injected at P0. Representative confocal images of whole-mount fluorescent immunolabeling of cochlea injected at P0 to illustrate the basal (a), middle (b), and apical turns (c). Ad-GFP-Baylor transduces supporting cells (SCs) and inner hair cells (IHCs) at basal (a), SCs at middle (b), and outer hair cells (OHCs) at apical turns (c). IHC: inner hair cell, OHC: outer hair cell, and SC: supporting cell. Scale bars: 10 *μ*m.

**Figure 3 fig3:**
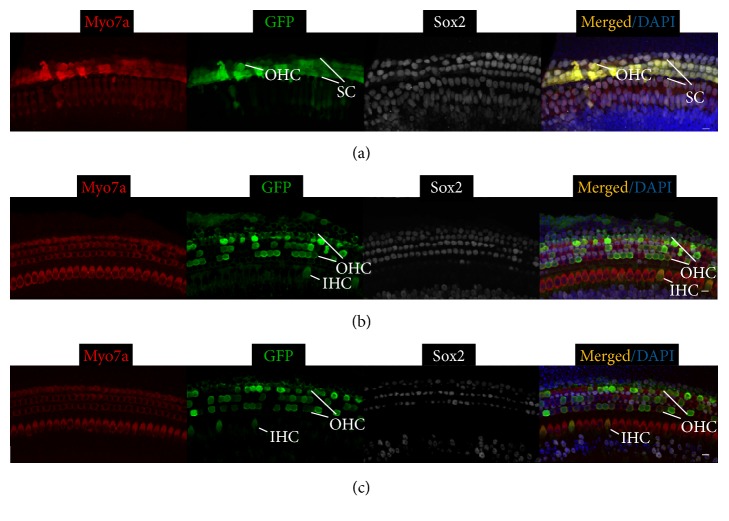
Ad-GFP-VB transduces diverse cell types in mouse cochlea when injected at P0. Representative confocal images of whole-mount fluorescent immunolabeling of the cochlea injected with adenovirus at P0 to illustrate the basal (a), middle (b), and apical turns (c). Ad-GFP-VB transduces SCs and OHCs at basal (a), OHCs, IHCs, and SCs at middle (b), and OHCs and IHCs at apical turns (c). IHC: inner hair cell, OHC: outer hair cell, and SC: supporting cell. Scale bars: 10 *μ*m.

**Figure 4 fig4:**
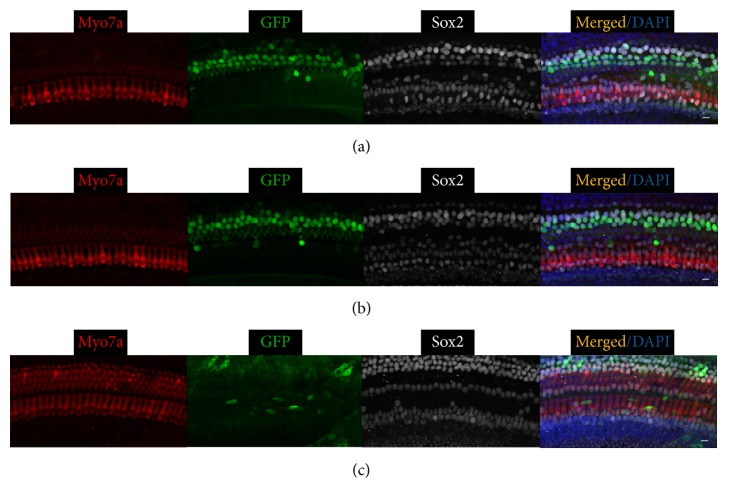
Ad-Cre-GFP-Baylor transduces supporting cells in the mouse cochlear when injected at P4. Representative confocal images of whole-mount fluorescent immunolabeling mouse cochlea to illustrate the basal (a), middle (b), and apical turns (c). Ad-Cre-GFP-Baylor transduces SCs at basal and middle turns efficiently. It transduces some SCs at the apical turn. Scale bars: 10 *μ*m.

**Figure 5 fig5:**
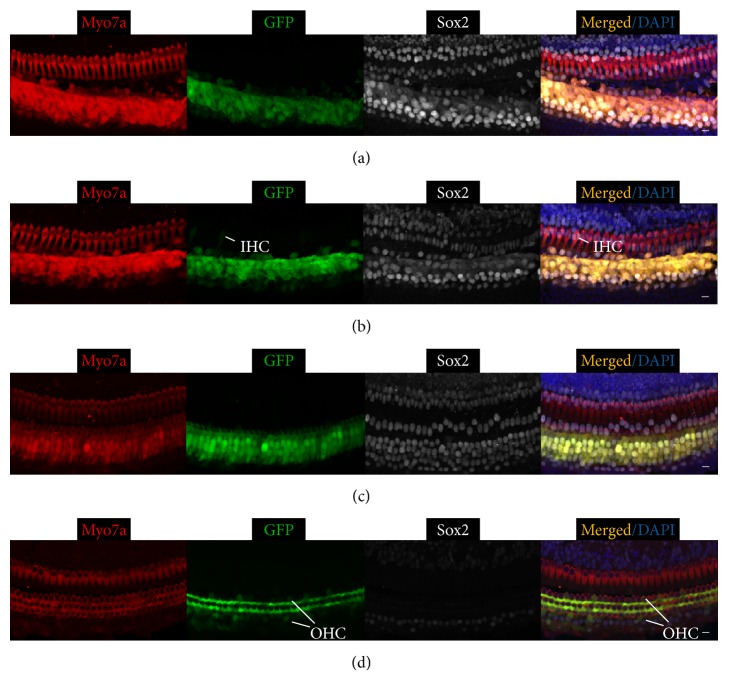
Ad-GFP-Baylor transduces mouse cochlea when injected at P4. Representative confocal images of whole-mount fluorescent immunolabeling P0 cochlea to illustrate the basal (a), middle (b), and apical turns (c, d). Ad-GFP-Baylor transduces SCs at basal (a), SCs and IHCs at middle (b), and SCs and OHCs at apical turns (c). Outer hair cells are transduced at the apical turn (d). IHC: inner hair cell; OHC: outer hair cell.

**Figure 6 fig6:**
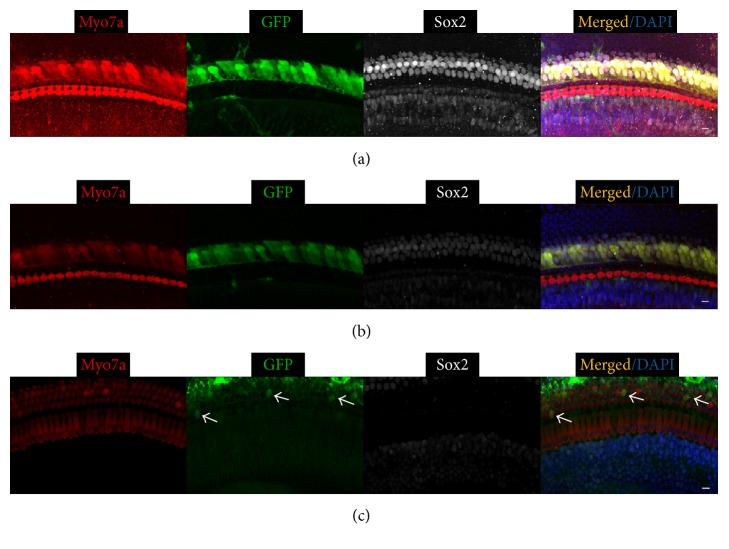
Ad-GFP-VB transduces supporting cells and OHCs in the mouse cochlear when injected at P4. Representative confocal images of whole-mount fluorescent immunolabeling of the cochlea to illustrate the basal (a), middle (b), and apical turns (c). Ad-GFP-VB transduces SCs at basal (a) and middle (b) and occasional OHCs at apical turns (arrows in (c)). Scale bars: 10 *μ*m.

**Figure 7:**
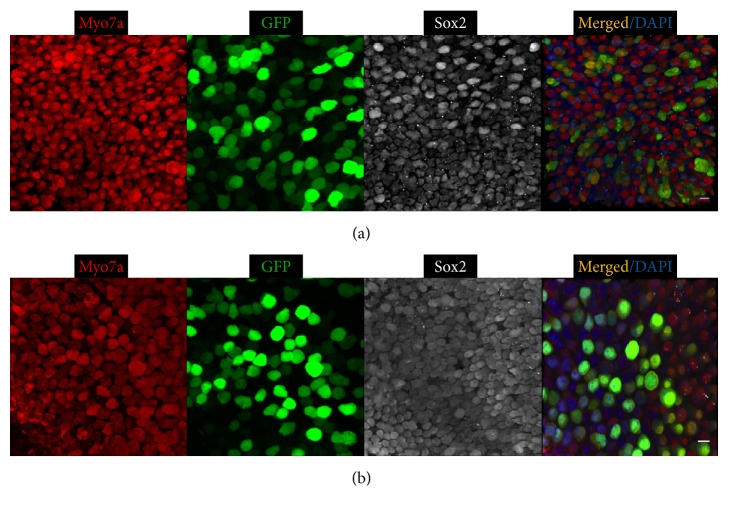
Ad-GFP-Baylor (a) and Ad-GFP-VB (b) transduce supporting cells and hair cells in the mouse utricle when injected at P0. Representative confocal images of whole-mount fluorescent immunolabeling of the utricle. Scale bars: 10 *μ*m.

We injected Ad vectors into the neonatal mouse inner ear at P0 or P4 via cochleostomy, because previous studies have shown that injection of AAV into the neonatal mouse cochlea by cochleostomy resulted in efficient transduction* in vivo* without adversely affecting hearing [8]. Four days after injection of any of the three Ad vectors into P0 or P4 mouse inner ears, cochlear structures remained intact and hair cells and supporting cells survived, indicating that the injection and Ad transduction did not damage inner ear cells (Figures 1 [Fig fig2] [Fig fig3] [Fig fig4] [Fig fig5] [Fig fig6]–7).

We assessed the transduction efficiency of the viral vectors by calculating the proportion of GFP positive cells, because all three Ad vectors carried the* GFP* gene. Whole-mount immunofluorescence labeling of HC and SC markers Myo7a and Sox2 identified hair cells and supporting cells, respectively. We found that the Ad-Cre-GFP-Baylor transduction was restricted to the injected side and no GFP expression was observed in the uninjected inner ear (Figure 1(d)). Same was true for Ad-GFP-Baylor and Ad-GFP-VB (data not shown). Our results confirmed that targeted delivery of Ad vectors via cochleostomy was confined within the injected inner ear.

Our results showed that three different Ad vectors injected at two different ages transduced different cell types in different cochlear regions with varying efficiencies (Table 1). Ad-Cre-GFP-Baylor, when injected at P0, efficiently transduced more than 70% of SCs but not HCs in the basal and middle turns (Figures 1(a) and 1(b)). It transduced few cells in the apical turn (Figure 1(c)). Comparing to injection at P0, injected at P4, Ad-Cre-GFP-Baylor transduced fewer SCs in the basal and middle turns, but more SCs in the apical turn (Figure 4).

A similar trend of transduction efficiency in SCs was observed for Ad-GFP-Baylor (Table 1). It transduced inner ear cells more efficiently than Ad-Cre-GFP-Baylor when injected at either P0 (Figure 2) or P4 (Figure 5). Furthermore, it also transduced a majority of OHCs in the apical turn and some IHCs in the basal turn when injected at P0 and IHCs in the middle turn and OHCs in the apical turn when injected at P4.

In contrast, Ad-GFP-VB transduced fewer SCs than either of the Ad-GFP-Baylor vectors with an opposite temporal trend: more efficiently when injected at P4 than at P0 (Table 1, Figures 3 and 6). Further, it more consistently transduced IHCs in the middle and apical turns and OHCs along the whole cochlear coil when injected at P0 (Table 1).

Ad-GFP-Baylor and Ad-GFP-VB transduced vestibular HCs and SCs with similar patterns when injected at either P0 or P4 (Figure 7), whereas Ad-Cre-GFP-Baylor transduced no utricular HCs or SCs.

In summary, we compared the transduction patterns of three Ad vectors injected at two different ages. All three Ad vectors consistently transduced SCs, with Ad-GFP-Baylor exhibiting the highest transduction rate across the whole cochlea. Ad-Cre-GFP-Baylor transduced only SCs in the basal and middle turns. Ad-GFP-Baylor and Ad-GFP-VB also transduced some HCs including both IHCs and OHCs (Table 1). The overall transduction efficiency was lower for Ad-GFP-Baylor vectors when injected at P4 than at P0 but higher for Ad-GFP-VB in SCs. Furthermore, Ad-GFP-VB had a broader transduction pattern when injected at P0 as it transduced OHCs along the whole cochlea and IHCs at middle and apex turns, but it only transduced a few OHCs at the apical turn when injected at P4.

## 4. Discussion

This study identified commercial adenovirus viral vectors that target mouse inner ear cell subtypes for gene delivery. Three adenovirus vectors transduced P0 and P4 inner ear, with different specificities and expression levels that are dependent on the type of adenoviral vectors and the age of mice. The cochlear sensory epithelium, which harbors auditory hair cells and supporting cells, is transduced with higher efficiency. The adenovirus with GFP alone transduced utricular supporting cells. The infected cells survived at the time of the study (four days after injection). The study shows that Ad vectors are capable of transducing mammalian inner ear efficiently and provides useful tools to evaluate gene therapy and to study inner ear gene function.

There are two approaches of hair cell regeneration: (1) direct transdifferentiation of surrounding cells especially supporting cells to change cell fate to become hair cells and (2) induction of cell cycle re-entry in cells such as supporting cells, which then further differentiate to replace damaged hair cells [1, 25, 26]. Thus, supporting cells are ideal candidates for hair cell regeneration by direct transdifferentiation or by renewed proliferation with subsequent transdifferentiation. Moreover, remaining hair cells can divide to generate new hair cells. Many cases of sensorineural hearing loss and vestibular dysfunction are caused by a primary pathology in the sensory epithelium [17, 18]. It is therefore important to express transgenes in the sensory epithelial cells such as supporting cells specifically. Three Ad vectors transduced SCs efficiently, an indication that they could be useful for potential hair cell regeneration studies. Ad-Cre-GFP-Baylor transduced SCs only at middle and base turns when injected at P0. The lack of transduction of the apical SCs was likely due to limited diffusion of the viral particles. Ad-Cre-GFP-Baylor transduces only SCs, whereas Ad-GFP-Baylor transduced 80% OHCs at apex turn and some IHCs at base turn at P0. Ad-GFP-VB transduces SCs and OHCs. Each Ad vector can therefore be selected to deliver genes to only SCs, HCs, or both.

The volume and titer of vector inoculation influence the cell types and location of cells transduced by the virus. To compare the difference of three virus infection, we use the same titer of three Ads. It is interesting that Ad-GFP-Baylor and Ad-Cre-GFP-Baylor had different transduction specificities. However, according to the instruction of Ad-GFP-Baylor from Baylor College of Medicine, the virus particle (vp) to plaque-forming unit (pfu) ratio is in a range of 1 : 10 to 1 : 200 and the titer is 2.5 × 10^10^–5 × 10^11^ pfu/ml. We used the titer of 10 × 10^10^ pfu/ml for dilution, which may be an underestimate of the actual titer as it had the highest transduction efficiency.

Injection into mouse cochlea through scala media by cochleostomy maximizes the efficiency as it allows virus to have access to many cochlear cell types. Previous studies suggested that, a better outcome of cell survival, mice younger than P5 should be used, as OHCs will generally die due to surgery in mice older than P5, especially at adult stage [8, 9, 13]. Future study needs to focus on identification of a route by which injection can be performed in adult without causing cell death or hearing loss.

Combining a therapeutic transgene with a reporter gene would be informative for easy identification of the cell types targeted. The human inner ear is much larger in size and would facilitate a more accurate delivery, which could help with the development of gene therapy in patients.

## 5. Conclusions

The present study explored commercial three Ad vectors into the mouse inner ear* in vivo*. The results show the feasibility of gene transfer into mouse inner ear via Ad vectors with different specificity and efficiency. Future application of gene delivery for the inner ear may include the induction of hair cell regeneration and treatment of hereditary deafness and vestibular dysfunction. The ability of the cochleostomy to deliver reporter transgenes into a variety of cell types in the inner ear, including the sensory epithelium, makes this method attractive to target inner ear cell subtypes. Continuous improvement in identification of highly efficient vectors targeting inner ear cell subtypes would advance their eventual use to treat hearing loss in human.

## Figures and Tables

**Table 1:** Comparison of *in vivo* transduction efficiency in different cell types and cochlear regions four days after injection of three adenoviral vectors into P0 or P4 mouse cochleae. *N* = 4 per condition. Apical: apical turn of the cochlea, basal: basal turn of the cochlea, IHCs: inner hair cells, middle: middle turn of the cochlea, OHCs: outer hair cells, P0: injected at postnatal day 0, P4: injected at postnatal day 4, and SCs: supporting cells.

Transduction efficiency (%)		IHCs			OHCs			SCs		
		Basal	Middle	Apical	Basal	Middle	Apical	Basal	Middle	Apical
Ad-Cre-GFP-Baylor	P0							74.5 ± 8.6	70.6 ± 7.8	
	P4							54.4 ± 6.8	52.4 ± 6.9	3.8 ± 1.2
Ad-GFP-Baylor	P0	9.1 ± 1.2					80.0 ± 12.0	90.8 ± 9.2	84.7 ± 10.6	
	P4		13.6 ± 2.9				17.4 ± 4.2	79.1 ± 10.6	74.3 ± 11.2	42.3 ± 6.1
Ad-GFP-VB	P0		9.1 ± 1.5	15.0 ± 4.8	9.8 ± 1.8	29.0 ± 4.9	27.8 ± 4.1	41.5 ± 8.1	7.7 ± 2.9	
	P4						2.9 ± 0.9	51.1 ± 6.2	33.1 ± 4.2	
